# 

**Published:** 1900-12-15

**Authors:** 


### ANNOUNCEMENTS.

### THE INSTITUTE OF DENTAL PEDAGOGICS.

The eighth annual meeting of this organization will be held December 27th, 28th and 29th, at the Maxwell House, in Nashville, Tenn. The following program has been prepared by the Executive Committee, and certainly presents sufficient variety and talent engaged to assure grand results. The time and place ought to assure a good attendance of all who are interested in dental educational affairs. The Executive Committee urge that at least one representative of each school be present:

THURSDAY, DEC. 27TH.

10:00 A. M.—Organization and Executive Business.

10:30 A. M.—President's Address, Dr. H. P. Carlton, San Francisco. Discussion by Drs. J. Taft, W. F. Litch, F. W. Weisse, H. B. Tileston, W. C. Barrett.

12:00 M.—The Use of Flexible Rubber in Orthodontia and Other Technic Teaching, by Dr. J. Q. Byram. Discussion by Drs. S. H. Guilford, C. S. Case, W. E. Graut, W. H. Fundenberg, W. W. Evans.

2:00 P. M.—Teaching of Materia Medica and Therapeutics, by D. A. H. Peck. Discussion by Drs. James Truman, Jno. I. Hart, S. W. Foster, G. E. Hunt, J. D. Patterson.

5:00 P. M.—Exhibits opened.

8:15 P. M.—The Use of the Lantern in Teaching Dental Histology in Its Relation to Operative Dentistry, by Dr. Fred. Noyes. Discussion by Drs. I. N. Bromell, A. H. Thompson, W. G. Foster, H. T. Smith, Louis Leroy.

FRIDAY, DEC. 28TH.

9:00 A. M.—Exhibit open.

10:00 A. M.—Presentation of the Technic of Crown and Bridge-work, Metal and Porcelain, by Dr. Thomas E. Weeks. Discussion by Drs. Otto Arnold, F. R. Sanduskey, R. H. Nones, R. H. Jewett, N. S. Hoff.

1:00 P. M.—Exhibit open.

2:00 P. M.—Class-room Methods of Teaching Oral Surgery, by Dr. G. V. I. Brown. Discussion by Drs. M. H. Cryer, T. S. Gilmer, Eugene Talbot, Ed. M. Kettig, J. Y. Crawford.

4:00 P. M.—A New Feature in Teaching Dental Anatomy and Operative Technics, by Dr. E. A. Webster. Discussion by Drs. E. C. Kirk, G. V. Black, Wm. A. Montell, G. W. Dittman, W. H. Whitslar.

SATURDAY, DEC. 29TH.

9:00 A. M.—Exhibit open.

10:00 A. M.—Class-room Methods of Teaching Prosthetic Dentistry, by Dr. Grant Molyneaux. Discussion by Drs. J. H. Kennerly, J. P. Gray, J. Bond Littig, A. O. Hunt, T. M. Allen.

11:30 A. M.—Report of Committees of Operative and Prosthetic Sylabii.

12:00 M.—Election of Officers.

